# Intrauterine fetal death due to *Brucella melitensis* infection: a case report and literature review

**DOI:** 10.1590/S1678-9946202567056

**Published:** 2025-08-18

**Authors:** Minxue Liu, Lina Zhou, Jiahui Liang, Linlin Li, Liying Huang, Huan Zhang

**Affiliations:** 1 Maternal and Child Health Hospital of Guangxi Zhuang Autonomous Region, Department of Laboratory Medicine, Nanning, Guangxi, People’s Republic of China; 2 Nanning Social Welfare Hospital, Guangxi, People’s Republic of China

**Keywords:** Intrauterine fetal death, Brucella melitensis, Brucellosis in pregnant women

## Abstract

Brucellosis is a zoonotic disease caused by *Brucella* species In pregnant women, this disease may lead to adverse obstetric outcomes, such as abortion, preterm delivery, intrauterine fetal death (IUFD), and congenital brucellosis. However, only a few studies have reported IUFD due to *Brucella*. In our case, one woman residing in Guangxi Province, China—a non-endemic region—developed symptoms 11 months after exposure. The disease manifested during her pregnancy—*Brucella melitensis* was identified via blood culture and confirmed by MALDI-TOF mass spectrometry. Despite receiving antibiotic treatment for brucellosis, she subsequently experienced IUFD. Additionally, we reviewed cases of IUFD secondary to brucellosis in the literature published until December 2024. Only three cases from the literature were enrolled in this study, which demonstrates that *Brucella*-related IUFD can occur in non-endemic zones, underscoring the need for increased clinical awareness.

## INTRODUCTION

Brucellosis is a zoonotic disease caused by the *Brucella* species and was first reported during pregnancy in 1908^1^. Abortion due to Brucellosis is more frequently reported in animals than in humans, so reports of adverse pregnancy outcomes in human brucellosis remain limited. The incidence in pregnant women ranges from 1.5% to 25% across regions^2-4^, and abortion is the most common adverse obstetric outcome, followed by preterm delivery, intrauterine fetal death (IUFD), and congenital brucellosis^5^. IUFD is defined as fetal death at ≥20 weeks gestation with weight ≥500 g^6^ and is a traumatic event for families. However, only a few studies of *Brucella*-associated IUFD have been reported. We report a case from Guangxi Province, China—a non-endemic region^7^—where IUFD occurred despite diagnosis and treatment. This study not only expands our knowledge of *Brucella*-associated IUFD but also provides evidence to guide prevention and clinical management in pregnant women.

### Ethics

Written informed consent was obtained from the patient for the publication of case details. This study was approved by the local Ethics Committee of The Maternal and Child Health Hospital of Guangxi Zhuang Autonomous Region.

## CASE REPORT

A 33-year-old 15 weeks pregnant woman showed up to the Maternal and Child Health Hospital of Guangxi Zhuang Autonomous Region (Nanning, China) with recurrent low-grade fever and vaginal spotting for two weeks. She had undergone an *in vitro* fertilization (IVF) process at another hospital 15 weeks prior, in which two embryos were transferred and one survived. After admission, her fever was 37.8 °C, and gynecological examination revealed that her cervical canal was shortened and dilated. Blood tests revealed a leukocyte count of 6.4 × 10^9^/L, a hemoglobin concentration of 106 g/L, and a platelet count of 165 × 10^9^/L. C-reactive protein was 31.93 mg/L, alanine aminotransferase 47 U/L, and the aspartate aminotransferase 71 U/L. Subsequently, examinations for *Mycobacterium tuberculosis*, *Mycoplasma pneumoniae,* and influenza virus yielded negative results. Two sets of blood cultures were obtained to evaluate suspected microorganism infection. She underwent cervical cerclage therapy on the second day of admission.

**Figure 1:**
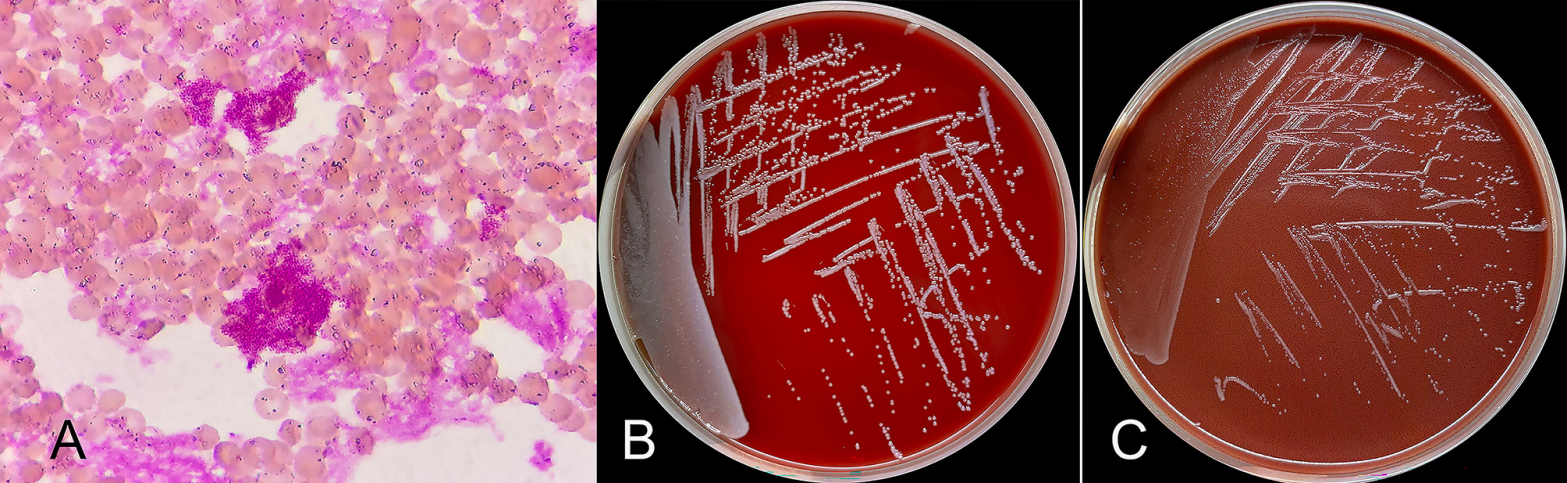
- The blood culture of a 33-year-old pregnant woman with recurrent low-grade fever and vaginal spotting: (A) Micromorphology examination showed Gram-negative coccobacilli, Gram stain, ×1000; (B, C) The colonies on blood agar plate and chocolate agar plate after three days incubated at 35 °C.

After 60 h, both aerobic blood cultures tested positive and microscopic examination revealed the presence of gram-negative coccobacilli (Figure 1A). However, the anaerobic blood culture remained negative after seven days of incubation. The clinical isolate was sub-cultured on blood agar plates and chocolate agar plates at 35 °C with 5% CO_2_, and after 72 hours, smooth, moist, opaque white colonies appeared (Figure 1B, 1C). The strain was identified as *B. melitensis* by Matrix-Assisted Laser Desorption/Ionization Time-of-Flight Mass Spectrometry (MALDI-TOF MS) (Antobio, China). Serum tube agglutination test for *Brucella* antigens yielded a ratio of 1:200. Further history revealed that the patient had been advised by an acquaintance that consuming sheep placenta could improve fertility, so 11 months prior to presentation she illegally purchased fresh sheep placentas twice. She reported barehanded contact with fresh sheep placentas during food preparation, with no other significant exposures in the preceding year.

**Figure 2:**
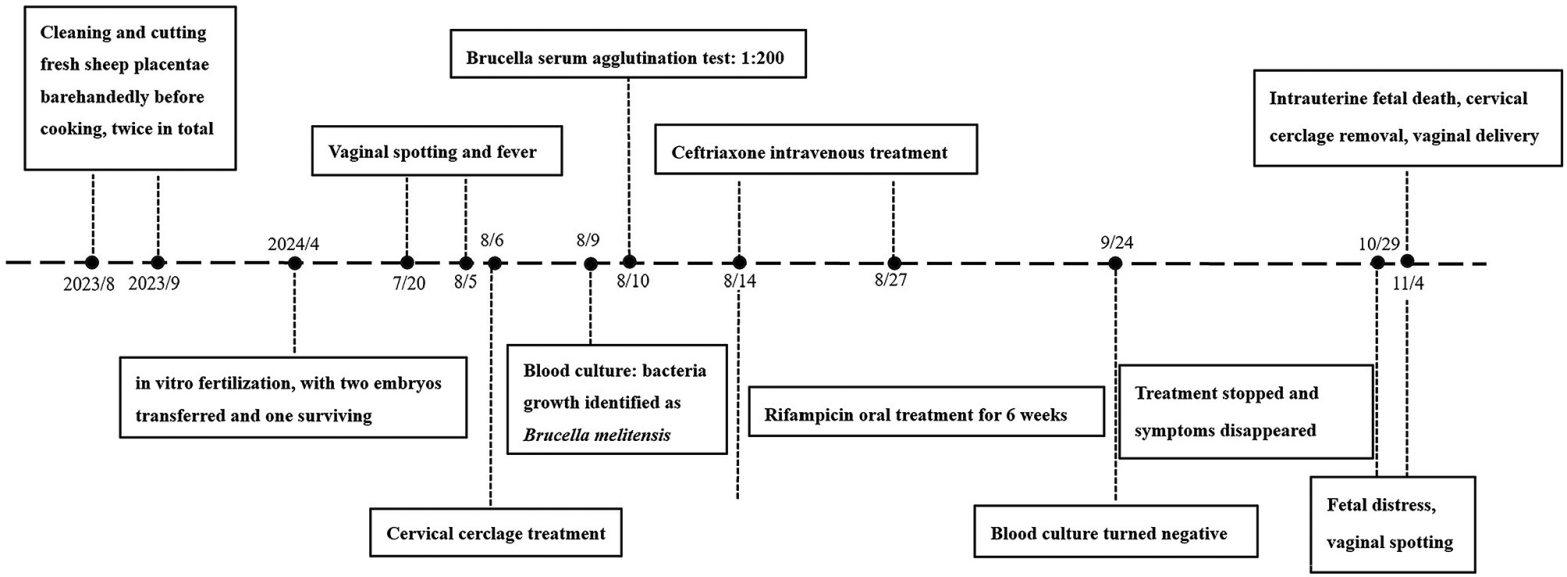
The onset and outcome of disease, diagnosis, and treatment.

The patient was diagnosed with brucellosis and was administered oral rifampicin (0.75g/day) and intravenous ceftriaxone (2 g/day) for 14 days. After her clinical condition improved she was discharged with a normal body temperature and negative antenatal examination. The patient continued taking oral rifampicin (0.75g/day) for more than four weeks, as recommended by the Chinese diagnosis and treatment plan for brucellosis^8^. She was asymptomatic, blood culture was negative, and antenatal examinations were normal from discharge to 30 weeks of gestation. At 34 weeks of gestation, the woman showed up to the hospital with vaginal spotting and a decrease in fetal movements over the previous four days. Upon examination, the patient was diagnosed with fetal distress. However, the patient and her family declined a cesarean section to terminate the pregnancy. Unfortunately, no fetal heart activity was detected on ultrasound 48 h later, and a dead male baby weighing 1500 g was delivered via provoked contractions. Pathological findings confirmed acute chorioamnionitis and funisitis. Three days after eutocia, the woman was asymptomatic and requested discharge, and no relapse was observed during the half-year follow-up period. Figure 2 shows the onset, diagnosis, treatment, and outcomes of the disease in this patient.

## DISCUSSION

China classifies brucellosis as a Class B infection, it spreads mainly through direct contact with infected animals, consumption of contaminated animal products, and inhaling aerosols^9^. Unlike the common transmission route in pregnant women—consumption of unpasteurized milk**—**as reported in the literature^10-13^, our patient acquired *B. melitensis by* handling infected sheep placentae during food preparation. Thus, health education is essential to interrupt brucellosis transmission routes.

Typically, brucellosis has an incubation period of one to four weeks, although some cases develop symptoms after up to one year. *Brucella* may remain in lymphoid tissues when the bacterial load is low, the strain’s virulence is weak, and host immunity is robust. Symptoms can emerge upon immune suppression, such as in our patient, who developed brucellosis during pregnancy. This infection often targets the placenta, which leads to adverse outcomes. A systematic review of 32 pregnant women across 10 countries reported that brucellosis caused preterm delivery (31.3%), abortion (37.5%), and IUFD (9.8%)^4^. Although IUFD accounts for fewer cases, it imposes greater economic and psychological burdens on patients and their families. Our patient had symptoms of threatened abortion at 15 weeks gestation. Following treatment with cervical cerclage and antibiotic therapy (ceftriaxone/rifampicin), her clinical symptoms improved, however, inflammation led to IUFD at 31 weeks gestation.

*Brucella* is an intracellular bacterium that poses therapeutic challenges. The World Health Organization (WHO) Expert Committee recommends treating brucellosis with a six-week course of doxycycline and rifampicin in non-pregnant patients^14^. For pregnant women, the recommended regimen is rifampicin plus trimethoprim-sulfamethoxazole, as tetracyclines may cause fetal tooth discoloration. However, trimethoprim-sulfamethoxazole is associated with neonatal kernicterus and its use is not recommended after 36 weeks of gestation. Supplementation with folinic acid should be administered if trimethoprim-sulfamethoxazole is used. Rifampicin is considered the safest antibiotic in pregnant women diagnosed with brucellosis^5^. In our case, despite adherence to the Chinese treatment protocol (two-week ceftriaxone plus six-week rifampicin), IUFD occurred at 31 weeks of gestation, 41 days after treatment completion. Thus, further investigation is required to know whether extended antibiotic therapy for *Brucella* infection could prevent adverse pregnancy outcomes. Additionally, the efficacy of combining rifampicin with trimethoprim-sulfamethoxazole for treating brucellosis during pregnancy, compared with monotherapy, also requires further evaluation.

Most patients with brucellosis manifest flu-like symptoms, including fever, headache, sweats, myalgia, fatigue, and arthralgia^9,13^. Pregnant women may also experience vaginal bleeding in the early disease stage^5^. Because of these nonspecific symptoms, brucellosis is often missed or misdiagnosed. Currently, the isolation of *Brucella* species from blood cultures is considered the “gold standard” for diagnosing brucellosis. *Brucella* species are strictly aerobic and fail to grow in anaerobic blood culture bottles, however, the small bacterial size and slow growth rate further complicate early laboratory identification. Additionally, serum agglutination test is a commonly used laboratory method for brucellosis diagnosis. Timely diagnosis is crucial for initiating appropriate treatment, particularly during pregnancy, to ensure favorable outcomes.

Here, we searched PubMed, Web of Science, Scopus and China National Knowledge Infrastructure (CNKI) or articles published until December 2024 reporting IUFD cases with documented Brucella infection. Case reports, series, and relevant reviews were included. After systematic screening, only three cases were documented (Table 1)^15-17^. Among these cases, one case was from Qinghai in northern China, a brucellosis-endemic region. Unlike these endemic cases, our patient was from Guangxi in southwestern China, a non-endemic area. Although human brucellosis cases have been reported in Guangxi^18^, our study presents the first documented case of Brucella-associated IUFD. In our case, antibiotic administration was delayed by 24 days after symptom onset. Despite receiving appropriate antibiotic treatment, IUFD ultimately occurred, consistent with the findings reported by Bosilkovski *et al*.^17^. To prevent adverse obstetric outcomes, early diagnosis, timely treatment, and proper infection management of brucellosis are essential.

**Table 1:** - Cases of intrauterine fetal death (IUFD) caused by *Brucella* reported in literature.

Article	Region	Age (years)	Diagnostic methods	Gestational weeks of IUFD	Clinical manifestations	Manner of transmission	Treatment	Time of IUFD occurrence	Treatment results	Pregnancy outcomes
Schreyer *et al.* ^15^	Israel	35	Blood culture	24	Fever, malaise, chills, nausea, lower abdominal pains	Not mentioned	Sodium cefalotin (4 g/day i.v.), oxytetracycline (500mg/day i.m.), cristalline penicillin (30 × l0^6^ i.v. for 1 week), gentamicin sulfate (3 × 80 mg i.m. for one week)	Prior to treatment	Clinical cure	IUFD
Dan *et al.* ^16^	Qinghai, China	27	ELISA	24	Fever, sweating, fatigue, general joints and muscles pain, insomnia	Not mentioned	Not mentioned	Prior to treatment	Clinical improvement	IUFD
Bosilkovski *et al.* ^17^	Macedonia	28	Rose Bengal, SAT	28	Fever, malaise, arthralgias, sweating	Direct contact with infected sheep	Rifampin plus cotrimoxazole (for 45 days, dosage not mentioned)	After treatment	Clinical cure	IUFD
This study	Guangxi, China	33	Blood culture, SAT	31	Fever, vaginal bleeding	Direct contact with infected sheep placentae	Ceftriaxone (2 g/day i.v. for 2 weeks), rifampicin (0.75g/day p.o. for 6 weeks)	After treatment	Clinical cure	IUFD

SAT = serum agglutination test; ELISA = enzyme-linked immunosorbent assay; IUFD = intrauterine fetal death; i.v. = intravenous; i.m. = intramuscular; p.o. = oral.

## CONCLUSIONS

To our knowledge, this represents the first documented case of IUFD caused by *B. melitensis* in a pregnant woman exposed to infected sheep placentae in Guangxi, China. Given the nonspecific clinical presentation, brucellosis diagnosis is frequently delayed in Guangxi, a non-endemic, poverty-stricken region of China. Physicians in maternity hospitals should maintain a high index of suspicion for brucellosis, because timely diagnosis, appropriate treatment, and effective infection control are essential to ensure favorable pregnancy outcomes.
